# The Impact of Lifestyle Interventions and Mental Health Management on Arrhythmias in Heart Failure

**DOI:** 10.1007/s11897-026-00789-2

**Published:** 2026-11-13

**Authors:** Jonathan P. Ariyaratnam, Adrian D. Elliott, Melissa E. Middeldorp, Prashanthan Sanders

**Affiliations:** 1 Centre for Heart Rhythm Disorders, Adelaide University and Royal Adelaide Hospital, Adelaide, Australia; 2 Department of Cardiology, University of Groningen, University Medical Center Groningen, Groningen, The Netherlands

## Abstract

**Purpose of Review:**

To summarise contemporary evidence on the impact of lifestyle interventions and mental health management on arrhythmias in patients with heart failure (HF), and to highlight emerging therapeutic opportunities.

**Recent Findings:**

Lifestyle modification targets shared risk factors in both arrhythmias and HF. In atrial fibrillation (AF), weight loss, exercise, alcohol reduction, and risk factor management reduce arrhythmia burden and improve arrhythmia-related symptoms and quality of life. Similarly, in both HF with reduced ejection fraction (HFrEF) and HF with preserved ejection fraction (HFpEF), exercise training improves functional capacity and quality of life, whilst dietary interventions and weight loss are particularly beneficial in HFpEF. Mental health disorders represent novel targets for intervention for arrhythmias in HF; these conditions are common in arrhythmia and HF patients and associated with poorer outcomes, but early data suggests that mental health interventions do not reduce arrhythmia burden or cardiovascular outcomes despite improving overall psychological well-being and quality of life.

**Summary:**

Lifestyle intervention to improve physical health is central to HF and arrhythmia care and established as a central pillar of management. Mental health represents an important but under-recognised target which requires urgent further investigation.

## Introduction

Cardiac arrhythmias are commonly associated with HF and have been shown to interact with both HF with reduced ejection fraction (HFrEF) and HF with preserved ejection fraction (HFpEF) in a bidirectional manner [1, 2]. Temporal data shows that the presence of HF increases the risk of both atrial and ventricular arrhythmias [3, 4], but it is also true that arrhythmias can induce the development of HF [5]. The link between HF and arrhythmias is underpinned by the presence of a group of shared cardiometabolic risk factors such as hypertension, diabetes and obesity, often developing as a result of unhealthy lifestyle habits including poor diet, excess alcohol consumption and physical inactivity [6].

Reversing these unhealthy lifestyle habits through lifestyle interventions represents an important management strategy for the effective treatment of both arrhythmias and HF [7–10]. Key lifestyle modification targets include lower calorie diets and weight loss, alcohol and smoking cessation (or reduction), increased physical activity and improved cardiorespiratory fitness as well as improved sleep quality. Indeed, comprehensive and structured lifestyle modification represents a key pillar in the guideline-directed management of both HF and atrial fibrillation (AF), the most common arrhythmia affecting patients with HF [11–13].

Whilst the evidence for lifestyle intervention to improve physical health in patients with arrhythmias and heart failure is strong, emerging evidence suggests that mental health may also be an important factor. Depression, anxiety and suicidal ideation is highly prevalent amongst patients with HF and arrhythmias and this is underrecognized by treating cardiologists and physicians [14]. There are several mechanisms by which mental health conditions could be linked with arrhythmias and heart failure, including autonomic dysfunction, the myocardial effects of cortisol and stress hormones and specific behaviours relating to medication adherence and lifestyle choices [15]. Mental health management could therefore represent a novel target for management of arrhythmias in heart failure.

Despite the evidence, lifestyle interventions to improve overall physical and mental health represent an underappreciated yet key factor to improve outcomes for patients with both arrhythmias and heart failure. The aim of this review is to summarize and highlight the key evidence to support lifestyle interventions in patients with arrhythmias and heart failure, focussing specifically on physical activity and exercise, diet and alcohol consumption as well as mental health management through mindfulness and stress reduction techniques, cognitive behavioural techniques and pharmacological therapies.

## Arrhythmias in Heart Failure

### Atrial Arrhythmias

Atrial arrhythmias, particularly AF, are highly prevalent in patients with heart failure and represent a major contributor to morbidity. AF affects approximately 20–40% of patients with heart failure [3] and real-world data suggests current incidence rates exceeding 200 cases per 1,000 person-years [16]. The burden of AF differs by heart failure phenotype, with particularly high prevalence in heart failure with preserved HFpEF, where rates often exceed those seen in HFrEF due to advanced age, atrial remodeling, and a higher burden of comorbidities such as hypertension and obesity [17]. Clinically, AF is associated significantly poorer outcomes in patients with both HFrEF and HFpEF, with increased risks of hospitalization, stroke, and mortality [18]. In addition, AF in HF is associated with worse symptoms, poorer exercise capacity and worse health-related quality of life [19].

Mechanistically, the presence of AF results in loss of atrial contraction and impaired ventricular filling. Indeed, cardioversion of AF to sinus rhythm has been shown to significantly increase cardiac output, both at rest and with exercise [20]. In HFpEF and HFrEF, therefore, the presence of AF likely has an impact on stroke volume and overall cardiac output. Indeed, AF ablation has been consistently shown to offer prognostic benefit in patients with HFrEF, although data remains limited in the HFpEF population at present [21].

### Ventricular Arrhythmias and Sudden Death

Patients with HF are also at significantly increased risk of ventricular arrhythmias and sudden cardiac death. In HFrEF, the risk of ventricular arrhythmias has been shown to be around 5% per year [22], whilst sudden cardiac death represents approximately 30–50% of all mortality in this population [23]. Implantable cardiac defibrillators (ICDs) now represent a cornerstone of management in both ischaemic and non-ischaemic HFrEF, having been shown to reduce mortality in selected HFrEF patients by up to 20–30% [22, 24]. In contrast to HFrEF, patients with HFpEF are at lower risk of ventricular arrhythmias and whilst sudden cardiac death remains the leading cause of death in HFpEF, observational data suggests that non-shockable rhythms such as asystole and pulseless electrical activity represent the primary mode of sudden death in HFpEF [25, 26]. Primary prevention ICDs are therefore not currently recommended in this cohort of patients.

Ventricular arrhythmias in heart failure arise from a combination of structural, electrical, and neurohormonal remodeling that together create an arrhythmogenic myocardial substrate. In heart failure with reduced ejection fraction (HFrEF), myocyte loss and replacement fibrosis (particularly in ischaemic cardiomyopathy) produce regions of slowed and heterogeneous conduction, enabling re-entry circuits leading to ventricular tachycardia [27]. In parallel, ion channel remodeling in HFrEF prolongs action potential duration and promotes triggered activity via early and delayed afterdepolarisations [28]. Elevated sympathetic tone and neurohormonal activation further increase automaticity and dispersion of repolarisation, lowering the threshold for ventricular fibrillation [29].

## Shared Risk Factors for the Development of Heart Failure and Arrhythmias

A significant reason for the close relationship between heart failure and arrhythmias is the shared risk factors underpinning the development of both conditions. These risk factors can be divided into non-modifiable and modifiable risk factors.

### Non-Modifiable Risk Factors

Non-modifiable risk factors play a fundamental role in predisposing individuals to both heart failure (HF) and arrhythmias. Advancing age is the most significant of these, associated with progressive myocardial fibrosis, reduced vascular compliance, and alterations in cardiac conduction. Specifically, these changes increase susceptibility to HFpEF, as well as atrial arrhythmias including AF [30]. Gender is also an important non-modifiable risk factor for both AF and HF; while men have a higher incidence of coronary artery disease, heart failure with reduced ejection fraction (HFrEF) and atrial arrhythmias, women are more likely to develop HFpEF [31].

Genetic predisposition further contributes, particularly through inherited cardiomyopathies and channelopathies, which can manifest as both myocardial dysfunction and malignant arrhythmias. Truncating variants in the TTN gene, which encodes titin, are a leading cause of dilated cardiomyopathy and are strongly associated with HFrEF as well as ventricular arrhythmias and sudden cardiac death [32]. Similarly, mutations in *LMNA* (lamin A/C) are particularly notable for their high arrhythmic burden, often leading to early conduction disease, ventricular arrhythmias, and progressive HF, with a high risk of sudden cardiac death even in the absence of severe systolic dysfunction [33]. Ion channel gene mutations, including those in *SCN5A*, which encodes the cardiac sodium channel, can produce overlapping phenotypes involving conduction disease, AF, ventricular arrhythmias, and cardiomyopathy [34]. Collectively, these genetic factors highlight the shared molecular pathways underpinning structural remodeling and electrical instability, reinforcing the close relationship between heart failure and arrhythmogenesis.

### Modifiable Risk Factors

Unlike non-modifiable risk factors, modifiable risk factors represent an important target for the treatment of both arrhythmias and HF. Several modifiable risk factors underlie the development of both arrhythmias and heart failure. Coronary artery disease, underpinned by common cardiovascular risk factors including hypercholesterolaemia, hypertension, diabetes and physical inactivity, plays a central role in the development ischaemic HFrEF and the consequent risk of ventricular arrhythmias and sudden cardiac death. Similarly, excess alcohol consumption is a critical risk factor in the development of both atrial and ventricular arrhythmias in certain non-ischaemic HFrEF phenotypes.

The close link between AF and HFpEF is also mediated by several shared risk factors. Perhaps the most important of these is obesity. Obesity exerts pleiotropic effects on cardiac structure and function, accelerating atrial cardiomyopathy as well as altering intracardiac haemodynamics through plasma volume expansion and cardiac enlargement secondary to ventricular hypertrophy and dilatation [35]. In addition, obesity is associated with increased volumes of epicardial adiposity, which increases the risk of AF through direct invasion into atrial myocardium and indirect paracrine effects leading to significant alterations in atrial electrophysiology. Greater epicardial fat volumes are also associated with increased risk of HFpEF, contributing to abnormal intracardiac haemodynamics primarily through increased pericardial restraint [35]. Another important risk factor is reduced cardiorespiratory fitness, which has been shown to increase left atrial stiffness and worsen left atrial mechanical and electrical function, leading to elevated left atrial pressures and resulting in the development of both AF and HFpEF [36]. Other traditional cardiac risk factors are also likely important contributors to the AF-HFpEF phenotype, including hypertension, diabetes mellitus, obstructive sleep apnoea and excess alcohol consumption [1].

## Lifestyle Interventions for Arrhythmias and HF

### Lifestyle Interventions for Arrhythmias

#### Atrial Arrhythmias

Lifestyle interventions play a central role in the management of atrial arrhythmias and this is reflected by its prominence as a central pillar in current guideline-recommended treatment of AF. Both observational data and randomized clinical trials demonstrate convincing evidence that modification of lifestyle through careful dietary restrictions alongside increased physical activity can reduce the recurrence of AF both before and after AF ablation procedures [6]. Weight loss represents an important target for lifestyle modifications in AF and several observational and randomized trials have confirmed this. The LEGACY-AF study showed that overweight patients achieving greater than 10% weight loss through dietary changes and increased physical activity demonstrated significant reductions in AF recurrences and AF symptom burden [9]. Similarly, in obsese patients with persistent AF, those who were able to achieve a ≥5 kg/m^2^ reduction in BMI through a supervised weight reduction program were more likely to remain free from AF recurrence after AF ablation, whilst weight loss through bariatric surgery has also demonstrated substantial benefits in AF patients [37].

Comprehensive lifestyle and risk factor modification programs targeting other risk factors in addition to weight loss have also demonstrated benefit in AF. The ARREST-AF observational cohort study and the subsequent ARREST-AF clinical trial showed that aggressive risk factor management, incorporating both lifestyle modifications and medical treatment of comorbidities, leading to reduced weight, improved blood pressure and glycaemic control reduced alcohol consumption (to three standard drinks per week) and treated obstructive sleep apnoea, significantly improved outcomes following AF ablation [7, 38]. The more recently published POP-AF trial similarly showed that a nurse-led integrated lifestyle clinic incorporating a home sleep apnoea test, weight reduction, alcohol reduction, smoking cessation, and optimal hypertension and hypercholesterolaemia treatment, significantly reduced hospitalisations for repeat ablations and direct current cardioversions [39].

Whilst data regarding comprehensive risk factor modification programs incorporating simultaneous management of all modifiable risk factors have shown consistent benefit in terms of AF outcomes, data regarding treatment of individual risk factors alone remains mixed. Reduction of alcohol consumption has perhaps demonstrated the greatest benefit, with a randomized study showing that complete abstinence from alcohol (rather than simply reduced alcohol intake) results in reduced AF recurrence [40]. The CARDIO-FIT study further demonstrated the benefit of improved cardiorespiratory fitness in AF, with patients who achieved greater cardiorespiratory gains exhibiting improved AF-free survival [41]. On the other hand, studies targeting improved blood pressure control alone have failed to demonstrate improved AF outcomes [42], whilst treatment of obstructive sleep apnoea with continuous positive airway pressure (CPAP) have also failed to deliver the expected benefits for AF patients, possibly due to poor treatment compliance or the selection of patients with more advanced atrial disease in these studies [43]. This data further highlights the importance of comprehensive lifestyle modifications as these changes provide the greatest opportunity to control all modifiable risk factors; improved diet and physical activity lead not only to weight loss but also an improvement in all other risk factors including obstructive sleep apnoea, blood pressure, glycaemia and cardiorespiratory fitness.

Mechanistically, weight loss has been shown to reduce and even reverse AF progression [44]. Animal studies show that this is underpinned by a reversal of the atrial substrate, with weight loss associated with reduced LA fibrosis, improved LA voltages and conduction velocities, reduced LA pressures and reduced AF inducibility [45]. Taken together, these data highlight the importance of lifestyle modifications to comprehensively manage and reverse the impact of the risk factors underlying AF.

#### Ventricular Arrhythmias

Whilst there is some evidence to support the incorporation of lifestyle modification in the management of ventricular arrhythmias, the evidence base is far less extensive than for AF. Unlike with AF, the best evidence comes from patients with obstructive sleep apnoea and heart failure, with CPAP treatment demonstrating a reduction in premature ventricular ectopics and non-sustained ventricular tachycardias [46–48]. In addition, there is some evidence to suggest that exercise training may reduce appropriate ICD shocks in patients with HFrEF at risk of ventricular arrhythmias [49]. However, there is limited evidence to support comprehensive risk factor modification as a method to reduce ventricular arrhythmias at this stage.

### Lifestyle Interventions for Heart Failure

Lifestyle management represents a core component of guideline-directed therapy in both HFrEF and HFpEF, regardless of the presence or absence of arrhythmias [50, 51]. Structured exercise training is strongly recommended and has been shown to improve functional status and quality of life in HFrEF and HFpEF [52]. In addition, there is evidence to suggest that exercise training may even have prognostic benefits [52, 53]. In HFpEF, exercise training has been shown to significantly improve peak oxygen consumption (VO_2Peak_), reduce heart failure symptoms and improve left ventricular diastolic function [54, 55]. Weight loss also represents an important target in HFpEF; an RCT showed that a 20-week program of caloric restriction alone resulted in a 7% weight loss and this led to an improvement in VO_2Peak_ of 1.3mL/kg [56]. Importantly, patients randomized to both caloric restriction and exercise demonstrated even greater improvements in exercise capacity, highlighting the additive effects of both diet and exercise in HFpEF patients. Dietary restriction and weight loss may be important in HFrEF although the evidence base is less strong [57]. Moderate salt restriction is also recommended in HFrEF, although a large randomized controlled trial found no evidence that a low-sodium diet significantly influenced HFrEF outcomes [58].

## Mental Health and Arrhythmias in Heart Failure

### Epidemiology

Emerging evidence suggests that mental health conditions are not only highly prevalent amongst patients with arrhythmias and heart failure but may be considered novel risk factors for the development of these conditions. In AF, the overall prevalence rates of depression and anxiety in patients with AF are 23.5% and 14.3% respectively with a preponderance in older patients and those of female gender [59]. More concerningly, it has been shown that severe psychological distress and even suicidal ideation are often present amongst patients with AF [14]. Additionally, further large scale epidemiological studies have shown that these mental health conditions may also increase the risk of AF development; population studies have demonstrated that depressed mood, exhaustion and post-traumatic stress disorders (PTSD) may all increase the risk of incident AF [14, 59, 60], suggesting a possible mechanistic role of these conditions in the pathophysiology of AF.

Similar associations have been demonstrated with ventricular arrhythmias. It is well-recognized that ventricular tachyarrhythmias and ICD shocks are associated with significant psychological impacts, including depression, anxiety and PTSD [61], but there is growing evidence to suggest that the relationship may be bidirectional. Depression has been associated with increased risk of future ventricular tachyarrhythmias, ICD shocks and sudden cardiac death in the general population as well as in patients with a prior diagnosis of HF [62]. Interestingly, during COVID-19 enforced social isolation measures, patients with ICDs demonstrated a 32% reduction in ventricular arrhythmias requiring device therapies, suggesting a potential role of real-life stressors (e.g. work-related stress) in triggering ventricular arrhythmias [63].

In HF, the prevalence of mental health disorders is even greater; 21.5% of patients with either HFrEF or HFpEF demonstrate clinically significant depressive symptoms, 19% meet criteria for major depressive disorder and 13% demonstrating generalised anxiety disorders [64]. Moreover, these conditions appear to worsen prognosis in HF with depressive disorders linked to increased risk of recurrent cardiac events, hospitalisations and even mortality. In patients without a baseline diagnosis of HF or cardiac disease, depression has been shown to increase the risk of HF development in subsequent years by up to 23% [65].

### Pathophysiological Mechanisms

**Fig. 1:**
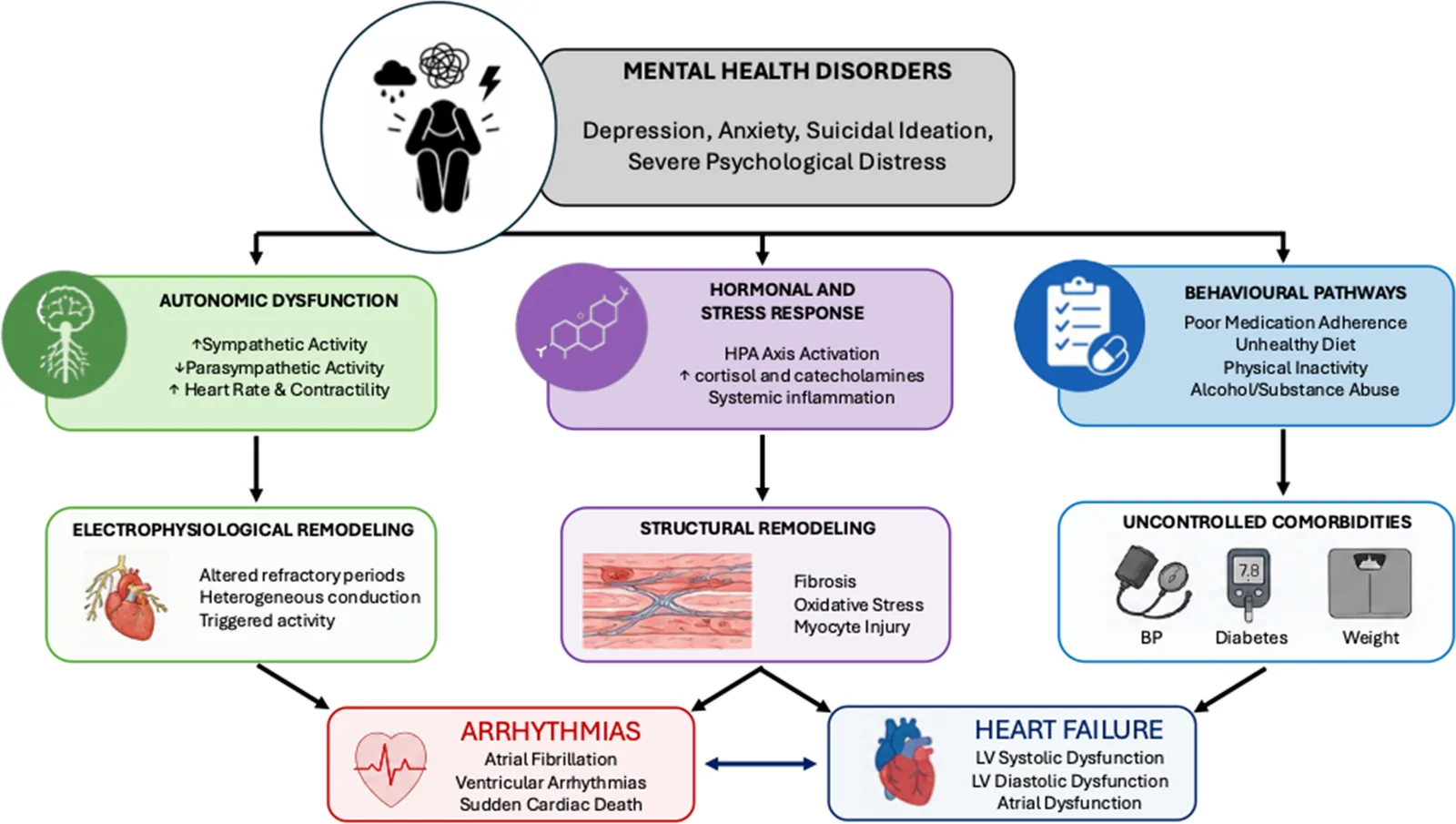
Pathophysiological mechanisms linking mental health disorders with arrhythmias and heart failure. Mental health disorders result in autonomic alterations, hormonal and stress responses and changes in behaviour, all of which are associated with structural and electrophysiological cardiac remodelling which can lead to the development of heart failure and arrhythmias

Figure 1 demonstrates the pathophysiological mechanisms underpinning the relationship between mental health conditions and the development of arrhythmias and heart failure. Mental health conditions contribute to the development of arrhythmias and heart failure through interconnected neurohormonal and autonomic pathways. Chronic psychological stress leads to autonomic imbalance, characterised by heightened sympathetic activation and reduced vagal tone, which promotes electrical instability and increases susceptibility to AF and ventricular arrhythmias. Activation of the hypothalamic–pituitary–adrenal axis results in elevated cortisol levels, while chronic systemic inflammation (e.g., increased cytokines such as IL-6 and TNF-α) contributes to adverse myocardial remodelling and fibrosis. These processes exacerbate structural and functional cardiac abnormalities, facilitating both arrhythmogenesis and progression of HF. Additionally, behavioural factors associated with mental health conditions, including reduced physical activity, poor medication adherence, and unhealthy lifestyle patterns, further compound cardiovascular risk.

### Mental Health Interventions and Evidence in Arrhythmias in Heart Failure

The evidence base for mental health interventions to treat patients with arrhythmias or HF continues to evolve and represents a potential novel therapeutic target. Table 1 provides a summary of the studies investigating the role of cognitive behavioural therapies on arrhythmia-related outcomes. Overall, whilst these therapies have consistently proven to improve overall quality of life and mental-health symptoms in arrhythmia patients, there is limited evidence to suggest these treatments can reduce arrhythmia burden or prognosis. In addition to cognitive-behavioural therapies, mind–body interventions have also shown promise; a small randomized study of yoga in patients with AF demonstrated significant reductions in AF episodes, symptom burden, and improvements in quality of life, likely mediated through favourable autonomic modulation [66].

**Table 1:** Mental health intervention studies in heart failure and arrhythmias

Study and Date	Country	Study Type	Number of Patients	Population	Follow-up duration	Intervention and Comparator	Outcomes
Atrial Fibrillation							
*Sårnholm* et al. 2023 [69]	Sweden	RCT	127	Symptomatic paroxysmal AF, no HF	3 months	10 week online AF-CBT program vs. standardized AF education	**↑** AF-specific QoL **↓** Healthcare consumption (56%) **→** AF burden
Malm et al. 2018	Sweden	RCT	111	AF, accompanied by spouse	12 months	Mindfulness-based CBT – three 2.5 h sessions over 9 weeks vs. treatment as usual	**↑** HRQoL **↓** Depression **↑** Sense of coherence
Shan et al. 2022 [70]	China	RCT	90	AF and depression diagnosis from psychiatrist	12 weeks	BraveHeart CBT (ten 1-hr sessions) vs. treatment as usual	**↑** HRQoL (SF12 - MCS) **↓** Depression (HAMD) **↓** Illness Perception (BIPQ)
Minje et al. 2023 [71]	China	Prospective, open, pseudorandomized	102	Paroxysmal AF	6 months	CBT vs. treatment as usual	**↑** HRQoL (SF12 - MCS) **↓** GAD7 **↓** BIPQ **↓** AFSS
ICD and Ventricular Arrhythmias							
Kohn et al. 2000 [72]	USA	RCT	49	Consecutive patients with ICD	9 months	CBT vs. no therapy	**↓** Depression **↓** Anxiety **↓** Psychological Distress **↑** Sexual functioning **→** Number of shocks
Chevalier et al. 2006 [73]	France	RCT	70	Secondary Prevention ICD patients	12 months	CBT vs. treatment as usual	**→** Number of shocks **↓** Anxiety **→** Depression **→** QoL
Lewin et al. 2009[74]	United Kingdom	RCT	192	Consecutive new ICD imaplants	6 months	Home-based cognitive behavioural rehabilitiation vs. usual care	**↑** HRQoL (SF12 - PCS) **↓** Depression **↓** Anxiety **↓** Unplanned admissions
Irvine et al. 2011[75]	Canada	RCT	193	New ICD Implant	12 months	CBT vs. usual cardiac care	**↓** Post-tramatic stress disorder **↓** Avoidance Symptoms **↓** Depression (women only) **↓** SF26 MCS (women only)
Russell et al. 2015 [76]	USA	RCT	83	3-months post ICD implant	6 months	10-week cognitive behavioural stress management vs. ‘Patient Education’	**↓** Tension/anxiety **↓** Anger **↓** Perceived stress BUT not sustained at 6 months
Berg et al. 2020 [77]	Denmark	RCT	88	ICD and Hospital Anxiety Depression Score (HADS)-Anxiety > 8 or HADS-Depression < 11	16 weeks	CBT+usual care vs. usual care	**↓** HADS-Anxiety **↓** HADS-Depression **↓** Becks Anxiety Inventory **↑** QoL (Global Heart QoL)

In HF, current guidelines recommend routine screening for depression and psychosocial distress, and emphasise referral for appropriate management, including psychological therapies and pharmacological treatment where indicated. However, these recommendations are based on expert consensus rather than strong outcome driven data. The SADHART-CHF trial demonstrated that pharmacotherapy with sertraline improved depressive symptoms but did not reduce mortality or hospitalisation [67]. More recent studies suggest that collaborative care models and structured psychological interventions may reduce depressive symptom burden and improve adherence and self-care behaviours [68]. Overall, current evidence suggests that whilst mental health interventions in the form of stress reduction techniques, CBT or pharmacotherapy may improve overall patient well-being, limited evidence exists to suggest that they can reduce arrhythmia burden or long-term hard cardiovascular outcomes.

## Conclusions

In conclusion, there is compelling evidence that lifestyle interventions represent a fundamental component of the management of patients with arrhythmias and heart failure, targeting the shared cardiometabolic substrates that underpin both conditions. Robust data support the role of weight loss, physical activity, alcohol moderation, and comprehensive risk factor management in improving outcomes, particularly in AF and HFpEF. In contrast, the evidence for ventricular arrhythmias remains more limited, although signals suggest benefit from selected interventions such as exercise and treatment of sleep-disordered breathing. Importantly, mental health conditions are highly prevalent and closely intertwined with both arrhythmogenesis and heart failure progression through complex neurohormonal, inflammatory, and behavioural mechanisms. While mental health interventions consistently improve quality of life and psychological wellbeing, their impact on hard cardiovascular endpoints remains uncertain. Future research should prioritise investigation of mental health interventions as a novel target for treatment.

## Key References

- Middeldorp ME, Ariyaratnam J, Lau D, Sanders P. Lifestyle modifications for treatment of atrial fibrillation. Heart. 2020;106(5):325-32.
  - ○ Narrative review of the evidence to support lifestyle modifications to improve outcomes in patients with atrial fibrillation.
- Pathak RK, Elliott AD, Lau DH, Middeldorp ME, Linz D, Fitzgerald JL, et al. Aggressive Risk Factor Reduction Study for Atrial Fibrillation Implications for Ablation Outcomes: The ARREST-AF Randomized Clinical Trial. JAMA Cardiol. 2025;10(12):1295-304.
  - ○ Randomized controlled trial demonstrating reduced AF recurrence with structured, comprehensive and aggressive risk factor modification in patients with AF.
- Kitzman DW, Brubaker P, Morgan T, Haykowsky M, Hundley G, Kraus WE, et al. Effect of Caloric Restriction or Aerobic Exercise Training on Peak Oxygen Consumption and Quality of Life in Obese Older Patients With Heart Failure With Preserved Ejection Fraction: A Randomized Clinical Trial. Jama. 2016;315(1):36-46.
  - ○ Randomized controlled trial demonstrating efficacy of caloric restriction and structured exercise program in improving outcomes in patients with heart failure with preserved ejection fraction.
- Walters TE, Wick K, Tan G, Mearns M, Joseph SA, Morton JB, et al. Psychological Distress and Suicidal Ideation in Patients With Atrial Fibrillation: Prevalence and Response to Management Strategy. J Am Heart Assoc. 2018;7(18):e005502.
  - ○ Observational study identifying a high prevalence of psychological distress and suicidal ideation amongst patients referred to cardiologists for management of atrial fibrillation.
- Särnholm J, Skúladóttir H, Rück C, Axelsson E, Bonnert M, Bragesjö M, et al. Cognitive Behavioral Therapy Improves Quality of Life in Patients With Symptomatic Paroxysmal Atrial Fibrillation. J Am Coll Cardiol. 2023;82(1):46-56.
  - ○ Randomised controlled trial showing that cognitive behavioural therapy can improve quality of life in patients with atrial fibrillation.

## Data Availability

No datasets were generated or analysed during the current study.

## References

[1] Ariyaratnam JP, Elliott AD, Mishima RS, Gallagher C, Lau DH, Sanders P. Heart failure with preserved ejection fraction: An alternative paradigm to explain the clinical implications of atrial fibrillation. Heart Rhythm O2. 2021;2(6):771–83. 10.1016/j.hroo.2021.09.015 PMC8710629 34988529

[2] Ariyaratnam JP, Lau DH, Sanders P, Kalman JM. Atrial Fibrillation and Heart Failure: Epidemiology, Pathophysiology, Prognosis, and Management. Card Electrophysiol Clin. 2021;13(1):47–62. 10.1016/j.ccep.2020.11.004 33516407

[3] Santhanakrishnan R, Wang N, Larson MG, Magnani JW, McManus DD, Lubitz SA, et al. Atrial fibrillation begets heart failure and vice versa. Circulation. 2016;133(5):484–92. 10.1161/CIRCULATIONAHA.115.018614 PMC4738087 26746177

[4] Cleland JG, Chattopadhyay S, Khand A, Houghton T, Kaye GC. Prevalence and incidence of arrhythmias and sudden death in heart failure. Heart Fail Rev. 2002;7(3):229–42. 10.1023/a:1020024122726 12215728

[5] Shoureshi P, Tan AY, Koneru J, Ellenbogen KA, Kaszala K, Huizar JF. Arrhythmia-induced cardiomyopathy. J Am Coll Cardiol. 2024;83(22):2214–32. 10.1016/j.jacc.2024.03.416 PMC13750622 38811098

[6] Middeldorp ME, Ariyaratnam J, Lau D, Sanders P. Lifestyle modifications for treatment of atrial fibrillation. Heart. 2020;106(5):325–32. 10.1136/heartjnl-2019-315327 31712316

[7] Pathak RK, Elliott AD, Lau DH, Middeldorp ME, Linz D, Fitzgerald JL, et al. Aggressive Risk Factor Reduction Study for Atrial Fibrillation Implications for Ablation Outcomes: The ARREST-AF Randomized Clinical Trial. JAMA Cardiol. 2025;10(12):1295–304. 10.1001/jamacardio.2025.4007 PMC12573115 41160038

[8] Pathak RK, Middeldorp ME, Lau DH, Mehta AB, Mahajan R, Twomey D, et al. Aggressive risk factor reduction study for atrial fibrillation and implications for the outcome of ablation: the ARREST-AF cohort study. J Am Coll Cardiol. 2014;64(21):2222–31. 10.1016/j.jacc.2014.09.028 25456757

[9] Pathak RK, Middeldorp ME, Meredith M, Mehta AB, Mahajan R, Wong CX, et al. Long-term effect of goal-directed weight management in an atrial fibrillation cohort. J Am Coll Cardiol. 2015;65(20):2159–69. 10.1016/j.jacc.2015.03.002 25792361

[10] Kitzman DW, Brubaker P, Morgan T, Haykowsky M, Hundley G, Kraus WE, et al. Effect of Caloric Restriction or Aerobic Exercise Training on Peak Oxygen Consumption and Quality of Life in Obese Older Patients With Heart Failure With Preserved Ejection Fraction. JAMA. 2016;315(1):36. 10.1001/jama.2015.17346 PMC4787295 26746456

[11] Joglar JA, Chung MK, Armbruster AL, Benjamin EJ, Chyou JY, Cronin EM, et al. 2023 ACC/AHA/ACCP/HRS Guideline for the Diagnosis and Management of Atrial Fibrillation: A Report of the American College of Cardiology/American Heart Association Joint Committee on Clinical Practice Guidelines. Circulation. 2024;149(1):e1–156. 10.1161/CIR.0000000000001193 PMC11095842 38033089

[12] McDonagh TA, Metra M, Adamo M, Gardner RS, Baumbach A, Böhm M, et al. 2021 ESC Guidelines for the diagnosis and treatment of acute and chronic heart failure. Eur Heart J. 2021;42(36):3599–726. 10.1093/eurheartj/ehab368 34447992

[13] Tzeis S, Gerstenfeld EP, Kalman J, Saad EB, Sepehri Shamloo A, Andrade JG et al. 2024 European Heart Rhythm Association/Heart Rhythm Society/Asia Pacific Heart Rhythm Society/Latin American Heart Rhythm Society expert consensus statement on catheter and surgical ablation of atrial fibrillation. Europace. 2024;26(4). 10.1093/europace/euae043 PMC11000153 38587017

[14] Walters TE, Wick K, Tan G, Mearns M, Joseph SA, Morton JB, et al. Psychological Distress and Suicidal Ideation in Patients With Atrial Fibrillation: Prevalence and Response to Management Strategy. J Am Heart Assoc. 2018;7(18):e005502. 10.1161/JAHA.117.005502 PMC6222970 30371197

[15] Peacock J, Whang W. Psychological distress and arrhythmia: risk prediction and potential modifiers. Prog Cardiovasc Dis. 2013;55(6):582–9. 10.1016/j.pcad.2013.03.001 PMC4014636 23621968

[16] Diaz J, Martinez F, Calderon JM, Fernandez A, Sauri I, Uso R, et al. Incidence and impact of atrial fibrillation in heart failure patients: real-world data in a large community. ESC Heart Fail. 2022;9(6):4230–9. 10.1002/ehf2.14124 PMC9773729 36111519

[17] Sartipy U, Dahlström U, Fu M, Lund LH. Atrial Fibrillation in Heart Failure With Preserved, Mid-Range, and Reduced Ejection Fraction. JACC Heart Fail. 2017;5(8):565–74. 10.1016/j.jchf.2017.05.001 28711451

[18] Khazanie P, Liang L, Qualls LG, Curtis LH, Fonarow GC, Hammill BG, et al. Outcomes of medicare beneficiaries with heart failure and atrial fibrillation. JACC Heart Fail. 2014;2(1):41–8. 10.1016/j.jchf.2013.11.002 PMC4174273 24622118

[19] Ariyaratnam JP, Elliott AD, Mishima RS, Kadhim K, McNamee O, Kuklik P, et al. Identification of subclinical heart failure with preserved ejection fraction in patients with symptomatic atrial fibrillation. JACC Heart Fail. 2023;11(11):1626–38. 10.1016/j.jchf.2023.07.019 37676212

[20] Morris JJ, Entman M, North WC, Kong Y, McIntosh H. The changes in cardiac output with reversion of atrial fibrillation to sinus rhythm. Circulation. 1965;31(5):670–8. 10.1161/01.cir.31.5.670 14278058

[21] Oraii A, McIntyre WF, Parkash R, Kowalik K, Razeghi G, Benz AP, et al. Atrial Fibrillation Ablation in Heart Failure With Reduced vs Preserved Ejection Fraction: A Systematic Review and Meta-Analysis. JAMA Cardiol. 2024;9(6):545–55. 10.1001/jamacardio.2024.0675 PMC11044015 38656292

[22] Bardy GH, Lee KL, Mark DB, Poole JE, Packer DL, Boineau R, et al. Amiodarone or an Implantable Cardioverter–Defibrillator for Congestive Heart Failure. N Engl J Med. 2005;352(3):225–37. 10.1056/NEJMoa043399 15659722

[23] Al-Khatib SM, Stevenson WG, Ackerman MJ, Bryant WJ, Callans DJ, Curtis AB, et al. 2017 AHA/ACC/HRS guideline for management of patients with ventricular arrhythmias and the prevention of sudden cardiac death. Circulation. 2017;138(13):CIR.00000000000. 10.1161/CIR.0000000000000549 29084731

[24] Moss AJ, Zareba W, Hall WJ, Klein H, Wilber DJ, Cannom DS, et al. Prophylactic Implantation of a Defibrillator in Patients with Myocardial Infarction and Reduced Ejection Fraction. N Engl J Med. 2002;346(12):877–83. 10.1056/NEJMoa013474 11907286

[25] Woolcott OO, Reinier K, Uy-Evanado A, Nichols GA, Stecker EC, Jui J, et al. Sudden cardiac arrest with shockable rhythm in patients with heart failure. Heart Rhythm. 2020;17(10):1672–8. 10.1016/j.hrthm.2020.05.038 PMC7541513 32504821

[26] Cho JH. Sudden death and ventricular arrhythmias in heart failure with preserved ejection fraction. Korean Circ J. 2022;52(4):251–64. 10.4070/kcj.2021.0420 PMC8989786 35388994

[27] Ebinger MW, Krishnan S, Schuger CD. Mechanisms of ventricular arrhythmias in heart failure. Curr Heart Fail Rep. 2005;2(3):111–7. 10.1007/s11897-005-0018-y 16138946

[28] Qu Z, Weiss JN. Mechanisms of ventricular arrhythmias: from molecular fluctuations to electrical turbulence. Annu Rev Physiol. 2015;77:29–55. 10.1146/annurev-physiol-021014-071622 PMC4342983 25340965

[29] Herring N, Kalla M, Paterson DJ. The autonomic nervous system and cardiac arrhythmias: current concepts and emerging therapies. Nat Rev Cardiol. 2019;16(12):707–26. 10.1038/s41569-019-0221-2 31197232

[30] Howie JO, Dziano JK, Ariyaratnam JP, Abbas M, Kenny GT, Evans S, et al. The association of age and left atrial dysfunction in patients with atrial fibrillation. Heart Rhythm. 2025;22(12):e1137–45. 10.1016/j.hrthm.2025.07.005 40651587

[31] Betai D, Ahmed AS, Saxena P, Rashid H, Patel H, Shahzadi A, et al. Gender Disparities in Cardiovascular Disease and Their Management: A Review. Cureus. 2024;16(5):e59663. 10.7759/cureus.59663 PMC11148660 38836150

[32] Johnson R, Fletcher RA, Peters S, Ohanian M, Soka M, Smolnikov A, et al. Titin-related familial dilated cardiomyopathy: factors associated with disease onset. Eur Heart J. 2025;46(48):5240–57. 10.1093/eurheartj/ehaf380 PMC12718689 40796136

[33] Khan SS, Castillo LM, Day SM. Prognostic Implications of Type and Location of LMNA Cardiomyopathy Genetic Variants. JAMA Cardiol. 2025;10(9):871–3. 10.1001/jamacardio.2025.2068 40601306

[34] Remme CA. SCN5A channelopathy: arrhythmia, cardiomyopathy, epilepsy and beyond. Philos Trans R Soc Lond B Biol Sci. 2023;378(1879):20220164. 10.1098/rstb.2022.0164 PMC10150216 37122208

[35] Ariyaratnam JP, Elliott AD, Mishima RS, Dziano JK, Emami M, Howie JO, et al. Evaluating the Impact of Obesity and Epicardial Adiposity on the Presence of HFpEF in Patients With AF. JACC Clin Electrophysiol. 2026;12(1):96–107. 10.1016/j.jacep.2025.08.011 40996395

[36] Ariyaratnam JP, Elliott AD, Mishima RS, Kadhim K, Emami M, Fitzgerald JL, et al. Structural, Functional and Electrical Remodelling of the Atria with Reduced Cardiorespiratory Fitness: Implications for AF. JACC Clin Electrophysiol; 2024. 10.1016/j.jacep.2024.05.014 38869505

[37] Donnellan E, Wazni OM, Kanj M, Baranowski B, Cremer P, Harb S, et al. Association between pre-ablation bariatric surgery and atrial fibrillation recurrence in morbidly obese patients undergoing atrial fibrillation ablation. EP Europace. 2019;21(10):1476–83. 10.1093/europace/euz183 31304532

[38] Pathak RK, Middeldorp ME, Lau DH, Mehta AB, Mahajan R, Twomey D, et al. Aggressive Risk Factor Reduction Study for Atrial Fibrillation and Implications for the Outcome of Ablation. J Am Coll Cardiol. 2014;64(21):2222–31. 10.1016/j.jacc.2014.09.028 25456757

[39] Vermeer J, Vinck-de Greef T, van den Broek M, de Louw B, van Steenbergen G, van Veghel D, et al. Improving outcomes of atrial fibrillation ablation by integrated personalized lifestyle interventions: a randomized controlled trial. Eur Heart J. 2026;47(6):669–79. 10.1093/eurheartj/ehaf689 40884040

[40] Voskoboinik A, Kalman JM, De Silva A, Nicholls T, Costello B, Nanayakkara S, et al. Alcohol abstinence in drinkers with atrial fibrillation. N Engl J Med. 2020;382(1):20–8. 10.1056/NEJMoa1817591 31893513

[41] Pathak RK, Elliott A, Middeldorp ME, Meredith M, Mehta AB, Mahajan R, et al. Impact of CARDIOrespiratory FITness on Arrhythmia Recurrence in Obese Individuals With Atrial Fibrillation: The CARDIO-FIT Study. J Am Coll Cardiol. 2015;66(9):985–96. 10.1016/j.jacc.2015.06.488 26113406

[42] Parkash R, Wells GA, Sapp JL, Healey JS, Tardif J-C, Greiss I, et al. Effect of aggressive blood pressure control on the recurrence of atrial fibrillation after catheter ablation. Circulation. 2017;135(19):1788–98. 10.1161/CIRCULATIONAHA.116.026230 28228428

[43] Hu J, Zuo S, Qian J, Cheng F, Wang D, Deng Y, et al. The effect of continuous positive airway pressure therapy on atrial fibrillation in patients with obstructive sleep apnea. Front Med (Lausanne). 2025;12:1509776. 10.3389/fmed.2025.1509776 PMC11810731 39935801

[44] Middeldorp ME, Pathak RK, Meredith M, Mehta AB, Elliott AD, Mahajan R, et al. PREVEntion and regReSsive effect of weight-loss and risk factor modification on atrial fibrillation: the REVERSE-AF study. EP Europace. 2018;20(12):1929–35. 10.1093/europace/euy117 29912366

[45] Mahajan R, Lau DH, Brooks AG, Shipp NJ, Wood JPM, Manavis J, et al. Atrial Fibrillation and Obesity: Reverse Remodeling of Atrial Substrate With Weight Reduction. JACC Clin Electrophysiol. 2021;7(5):630–41. 10.1016/j.jacep.2020.11.015 33640353

[46] Domaradzki D, Lelakowski J, Konieczyńska M, Matusik PT. Continuous positive airway pressure treatment reduces ventricular arrhythmias in obstructive sleep apnea patients with nocturnal dominance of arrhythmias and severe desaturations. Pol Arch Intern Med. 2022;132(6):16236. 10.20452/pamw.16236. 35362714

[47] Ryan CM, Usui K, Floras JS, Bradley TD. Effect of continuous positive airway pressure on ventricular ectopy in heart failure patients with obstructive sleep apnoea. Thorax. 2005;60(9):781–5. 10.1136/thx.2005.040972 PMC1747520 15994252

[48] Seyis S, Usalan AK, Rencuzogullari I, Kurmuş Ö, Gungen AC. The effects of continuous positive airway pressure on premature ventricular contractions and ventricular wall stress in patients with heart failure and sleep apnea. Can Respir J. 2018. 10.1155/2018/2027061. PMC5829433 29623136

[49] Pandey A, Parashar A, Moore C, Ngo C, Salahuddin U, Bhargava M, et al. Safety and Efficacy of Exercise Training in Patients With an Implantable Cardioverter-Defibrillator: A Meta-Analysis. JACC Clin Electrophysiol. 2017;3(2):117–26. 10.1016/j.jacep.2016.06.008 29759383

[50] McDonagh TA, Metra M, Adamo M, Gardner RS, Baumbach A, Böhm M, et al. 2021 ESC Guidelines for the diagnosis and treatment of acute and chronic heart failure. Eur Heart J. 2021;42(36):3599–726. 10.1093/eurheartj/ehab368 34447992

[51] Heidenreich PA, Bozkurt B, Aguilar D, Allen LA, Byun JJ, Colvin MM, et al. 2022 AHA/ACC/HFSA Guideline for the Management of Heart Failure: A Report of the American College of Cardiology/American Heart Association Joint Committee on Clinical Practice Guidelines. Circulation. 2022;145(18):e895–1032. 10.1161/CIR.0000000000001063 35363499

[52] O’Connor CM, Whellan DJ, Lee KL, Keteyian SJ, Cooper LS, Ellis SJ, et al. Efficacy and safety of exercise training in patients with chronic heart failure: HF-ACTION randomized controlled trial. JAMA. 2009;301(14):1439–50. 10.1001/jama.2009.454 PMC2916661 19351941

[53] Piepoli MF, Davos C, Francis DP, Coats AJ. Exercise training meta-analysis of trials in patients with chronic heart failure (ExTraMATCH). BMJ. 2004;328(7433):189. 10.1136/bmj.37938.645220.EE PMC318480 14729656

[54] Edelmann F, Wachter R, Duvinage A, Mueller S, Fegers-Wustrow I, Schwarz S, et al. Combined endurance and resistance exercise training in heart failure with preserved ejection fraction: a randomized controlled trial. Nat Med. 2025;31(1):306–14. 10.1038/s41591-024-03342-7 PMC11750725 39747684

[55] Edelmann F, Gelbrich G, Düngen H-D, Fröhling S, Wachter R, Stahrenberg R, et al. Exercise Training Improves Exercise Capacity and Diastolic Function in Patients With Heart Failure With Preserved Ejection Fraction: Results of the Ex-DHF (Exercise training in Diastolic Heart Failure) Pilot Study. J Am Coll Cardiol. 2011;58(17):1780–91. 10.1016/j.jacc.2011.06.054 21996391

[56] Kitzman DW, Brubaker P, Morgan T, Haykowsky M, Hundley G, Kraus WE, et al. Effect of Caloric Restriction or Aerobic Exercise Training on Peak Oxygen Consumption and Quality of Life in Obese Older Patients With Heart Failure With Preserved Ejection Fraction: A Randomized Clinical Trial. JAMA. 2016;315(1):36–46. 10.1001/jama.2015.17346 PMC4787295 26746456

[57] Khan MS, Javaid SS, Petrie MC, Zieroth S, Anker SD, Butler J. Obesity and Heart Failure With Reduced Ejection Fraction. A Review. JACC; 2026. 10.1016/j.jacc.2026.02.5083 41949514

[58] Ezekowitz JA, Colin-Ramirez E, Ross H, Escobedo J, Macdonald P, Troughton R, et al. Reduction of dietary sodium to less than 100 mmol in heart failure (SODIUM-HF): an international, open-label, randomised, controlled trial. Lancet. 2022;399(10333):1391–400. 10.1016/S0140-6736(22)00369-5 35381194

[59] Zhang S, Zhang N, Liu L, Zheng W, Ma ZL, Qiao SY, et al. Global epidemiology of mental disorder in atrial fibrillation between 1998–2021: a systematic review and meta-analysis. World J Psychiatry. 1998;14(1):179–93. 10.5498/wjp.v14.i1.179 PMC10845231 38327890

[60] Garg PK, Claxton JS, Soliman EZ, Chen LY, Lewis TT, Mosley T, et al. Associations of anger, vital exhaustion, anti-depressant use, and poor social ties with incident atrial fibrillation: the Atherosclerosis Risk in Communities Study. Eur J Prev Cardiol. 2021;28(6):633–40. 10.1177/2047487319897163 PMC8981832 34021575

[61] Gupta K, Kaminska M, Gupta S, Waraich H, Malik S, Meghdadi A, et al. The Psychological Impact of Implantable Cardioverter Defibrillators: A Narrative Review. Am J Cardiol. 2025;255:83–8. 10.1016/j.amjcard.2025.07.007 40659282

[62] You Y, Shi Y, Yu Q, Rao X, Tong X, Tang T, et al. Depression and risk of sudden cardiac death and arrhythmias: a systematic review and meta-analysis. Rev Cardiovasc Med. 2025;26(8):36520. 10.31083/RCM36520 PMC12415728 40927095

[63] O’Shea CJ, Thomas G, Middeldorp ME, Harper C, Elliott AD, Ray N, et al. Ventricular arrhythmia burden during the coronavirus disease 2019 (COVID-19) pandemic. Eur Heart J. 2021;42(5):520–8. 10.1093/eurheartj/ehaa893 PMC7953962 33321517

[64] Rutledge T, Reis VA, Linke SE, Greenberg BH, Mills PJ. Depression in heart failure a meta-analytic review of prevalence, intervention effects, and associations with clinical outcomes. J Am Coll Cardiol. 2006;48(8):1527–37. 10.1016/j.jacc.2006.06.055 17045884

[65] Cao L, Sheng C, Luo G, Ou J. Depression as a Risk Factor for Developing Heart Failure: A Meta-Analysis of Prospective Cohort Studies. J Cardiovasc Nurs. 2022;37(2):112–21. 10.1097/JCN.0000000000000760 33148960

[66] Lakkireddy D, Atkins D, Pillarisetti J, Ryschon K, Bommana S, Drisko J, et al. Effect of yoga on arrhythmia burden, anxiety, depression, and quality of life in paroxysmal atrial fibrillation: the YOGA My Heart Study. J Am Coll Cardiol. 2013;61(11):1177–82. 10.1016/j.jacc.2012.11.060 23375926

[67] O’Connor CM, Jiang W, Kuchibhatla M, Silva SG, Cuffe MS, Callwood DD, et al. Safety and efficacy of sertraline for depression in patients with heart failure: results of the SADHART-CHF (Sertraline Against Depression and Heart Disease in Chronic Heart Failure) trial. J Am Coll Cardiol. 2010;56(9):692–9. 10.1016/j.jacc.2010.03.068 PMC3663330 20723799

[68] Freedland KE, Skala JA, Carney RM, Steinmeyer BC, Rubin EH, Rich MW. Sequential Interventions for Major Depression and Heart Failure Self-Care: A Randomized Clinical Trial. Circulation: Heart Fail. 2022;15(8):e009422. 10.1161/CIRCHEARTFAILURE.121.009422 PMC9389592 35973032

[69] Särnholm J, Skúladóttir H, Rück C, Axelsson E, Bonnert M, Bragesjö M, et al. Cognitive Behavioral Therapy Improves Quality of Life in Patients With Symptomatic Paroxysmal Atrial Fibrillation. J Am Coll Cardiol. 2023;82(1):46–56. 10.1016/j.jacc.2023.04.044 37380303

[70] Shan Q, Xinxin S, Zhijuan X, Rongjing D, Minjie Z. Effects of Cognitive Behavior Therapy on Depression, Illness Perception, and Quality of Life in Atrial Fibrillation Patients. Front Psychiatry. 2022;13:830363. 10.3389/fpsyt.2022.830363 PMC9120611 35599772

[71] Minjie Z, Zhijuan X, Xinxin S, Xinzhu B, Shan Q. The effects of cognitive behavioral therapy on health-related quality of life, anxiety, depression, illness perception, and in atrial fibrillation patients: a six-month longitudinal study. BMC Psychol. 2023;11(1):431. 10.1186/s40359-023-01457-z PMC10704769 38062475

[72] Kohn CS, Petrucci RJ, Baessler C, Soto DM, Movsowitz C. The effect of psychological intervention on patients’ long-term adjustment to the ICD: a prospective study. Pacing Clin Electrophysiol. 2000;23(4 Pt 1):450–6. 10.1111/j.1540-8159.2000.tb00826.x 10793433

[73] Chevalier P, Cottraux J, Mollard E, Sai N, Brun S, Burri H, et al. Prevention of implantable defibrillator shocks by cognitive behavioral therapy: a pilot trial. Am Heart J. 2006;151(1):191. 10.1016/j.ahj.2005.10.007 16368316

[74] Lewin RJ, Coulton S, Frizelle DJ, Kaye G, Cox H. A brief cognitive behavioural preimplantation and rehabilitation programme for patients receiving an implantable cardioverter-defibrillator improves physical health and reduces psychological morbidity and unplanned readmissions. Heart. 2009;95(1):63–9. 10.1136/hrt.2007.129890 18070951

[75] Irvine J, Firestone J, Ong L, Cribbie R, Dorian P, Harris L, et al. A randomized controlled trial of cognitive behavior therapy tailored to psychological adaptation to an implantable cardioverter defibrillator. Psychosom Med. 2011;73(3):226–33. 10.1097/PSY.0b013e31820afc63 21321256

[76] Russell DC, Smith TL, Krahn DD, Graskamp P, Singh D, Kolden GG, et al. Effects of Cognitive Behavioral Stress Management on Negative Mood and Cardiac Autonomic Activity in ICD Recipients. Pacing Clin Electrophysiol. 2015;38(8):951–65. 10.1111/pace.12668 26010524

[77] Berg SK, Rasmussen TB, Herning M, Svendsen JH, Christensen AV, Thygesen LC. Cognitive behavioural therapy significantly reduces anxiety in patients with implanted cardioverter defibrillator compared with usual care: findings from the Screen-ICD randomised controlled trial. Eur J Prev Cardiol. 2020;27(3):258–68. 10.1177/2047487319874147 31575299

